# Supplementary material for: Dietary Strategies by Foods with Antioxidant Effect on Nutritional Management of Dyslipidemias: A Systematic Review
Source: Antioxidants (Basel). 2021 Feb 3;10(2):225. doi: 10.3390/antiox10020225 (PMC7913156; doi:10.3390/antiox10020225)
Supplement: Supplementary file 1 [file antioxidants-10-00225-s001.zip › Supplementary files/Figure S1.pdf]

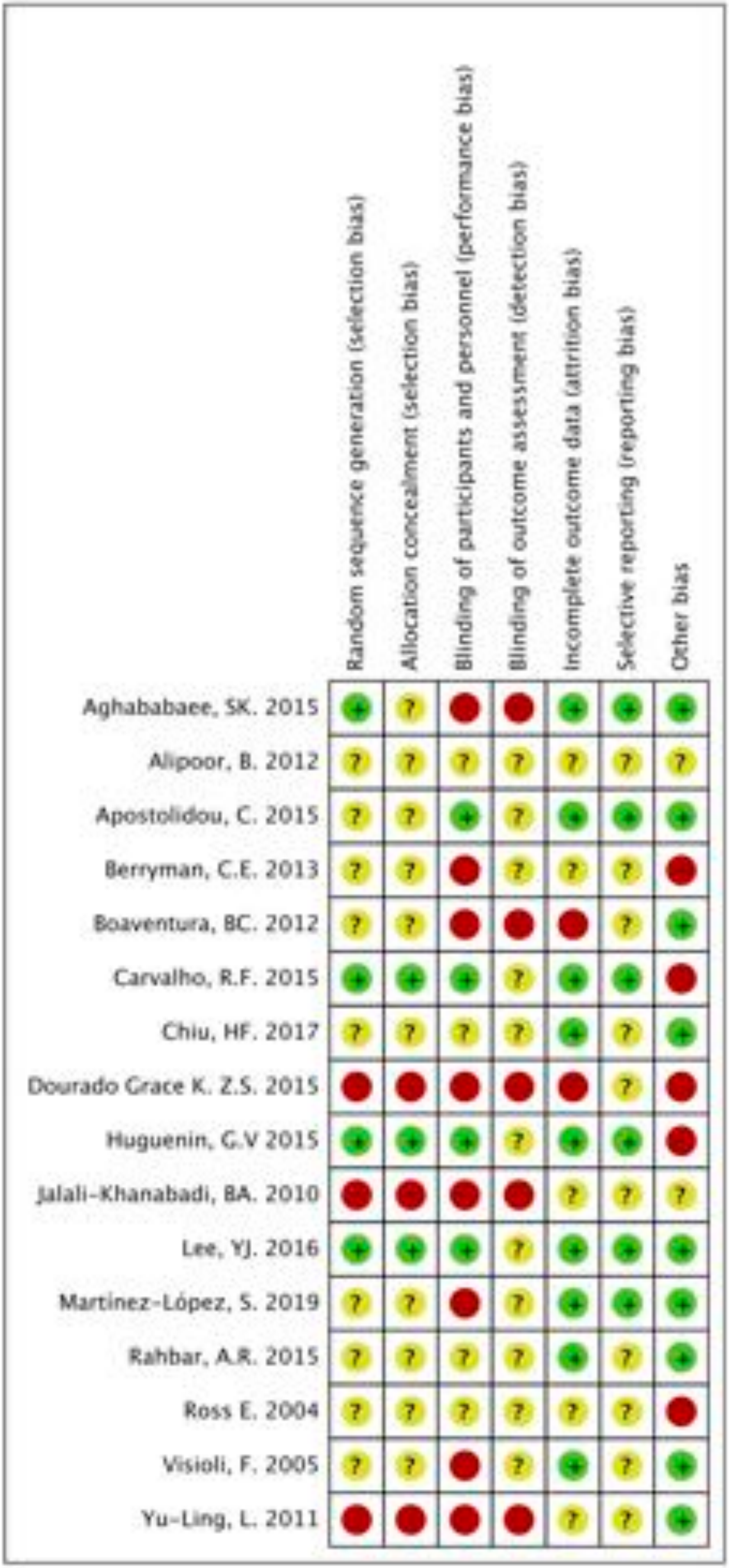
Random sequence generation (selection bias)
Allocation concealment (selection bias)
Blinding of participants and personnel (performance bias)
Blinding of outcome assessment (detection bias)
Incomplete outcome data (attrition bias)
Selective reporting (reporting bias)
Other bias
Aghababaee, SK. 2015
Alipoor, B. 2012
Apostolidou, C. 2015
Berryman, C.E. 2013
Boaventura, BC. 2012
Carvalho, R.F. 2015
Chiu, HF. 2017
Dourado Grace K. Z.S. 2015
Huguenin, G.V 2015
Jalali-Khanabadi, BA. 2010
Lee, YJ. 2016
Martinez-López, S. 2019
Rahbar, A.R. 2015
Ross E. 2004
Visioli, F. 2005
Yu-Ling, L. 2011
