# Supplementary material for: Dietary Strategies by Foods with Antioxidant Effect on Nutritional Management of Dyslipidemias: A Systematic Review
Source: Antioxidants (Basel). 2021 Feb 3;10(2):225. doi: 10.3390/antiox10020225 (PMC7913156; doi:10.3390/antiox10020225)
Supplement: Supplementary file 1 [file antioxidants-10-00225-s001.zip › Supplementary files/Table S1.docx]

*Table S1.* **Quality assessment based on the CONSORT 2010 checklist of the articles included.**

| **Author** | **Year** | **Items reported, total reported (%)** | **Items partially reported, total partially reported (%)** | **Items not reported, total not reported (%)** | **Not applicable items, total (%)** |
| --- | --- | --- | --- | --- | --- |
| Ros, E. | 2004 | 1a, 1b, 2a, 2b, 3a, 4a, 4b, 5, 6a, 7a, 11b, 12a, 12b, 13a, 13b, 14a, 14b, 15, 17a, 18, 20, 21, 22, 25 **(64.8%)** |  | 8a, 8b, 9, 10, 11a, 16, 19, 23, 24 **(24.3%)** | 3b, 6b, 7b, 17b **(10.8%)** |
| Visioli, F. | 2005 | 1b, 2a, 2b, 3a, 4a, 4b, 5, 6a, 11b, 14a, 14b, 16, 17a, 21, 22, 25 **(43.2%)** |  | 1a, 7a, 8a, 8b, 9, 10, 11a, 12a, 12b, 13a, 13b, 15, 18, 19, 20, 23, 24 **(46%)** | 3b, 6b, 7b, 17b **(10.8%)** |
| Jalali-Khanabadi, B.A. | 2010 | 1b, 2a, 2b, 4a, 5, 6a, 12a, 14b, 17a, 21, 22, 25 **(32.4%)** | 4b **(2.7%)** | 1a, 7a, 8a, 8b, 9, 10, 11a, 11b, 12b, 13a, 13b, 14a, 15, 16, 19, 20, 23, 24 **(48.4 %)** | 3a, 3b, 6b, 7b, 17b, 18 **(16.2%)** |
| Yu-Ling, L. | 2011 | 1b, 2a, 2b, 3a, 4a, 4b, 5, 6a, 12a, 13b, 14a, 14b, 15, 16, 17a, 19, 21, 22, 25 **(51.4%)** | 13a **(2.7%)** | 1a, 7a, 8a, 8b, 9, 10, 11a, 11b, 12b, 18, 20, 23, 24 **(35.1%)** | 3b, 6b, 7b, 17b **(10.8%)** |
| Alipoor, B. | 2012 | 1b, 2a, 2b, 3a, 4a, 4b, 5, 6a, 7a, 7b, 12a, 12b, 14a, 14b, 17a, 18, 21, 22, 25 **(51.4%)** | 23,24 **(5.4%)** | 1a, 8a, 8b, 9, 10, 11a, 11b, 13a, 13b, 15, 16, 19, 20 **(35.1%)** | 3b, 6b, 17b **(8.1%)** |
| Boaventura, B.C. | 2012 | 1b, 2a, 2b, 3a, 4a, 5, 6a, 11b, 12a, 13a, 13b, 14a, 14b, 15, 16, 17a, 19, 21, 22, 25 **(54.1%)** | 4b **(2.7%)** | 1a, 8a, 8b, 9, 10, 11a, 20, 23, 24 **(24.3%)** | 3b, 6b, 7a, 7b, 12b, 17b, 18 **(18.9%)** |
| Berryman, C.E. | 2013 | 1b, 2a, 2b, 3a, 4a, 4b, 5, 6a, 7a, 11b, 12a, 12b, 14a, 14b, 15, 17a, 18, 19, 20, 21, 22, 25 **(59.5%)** | 16 **(2.7%)** | 1a, 8a, 8b, 9, 10, 11a, 13a, 13b, 23, 24 **(27%)** | **3b, 6b, 7b, 17b (10.8%)** |
| Carvalho, R.F. | 2015 | 1a, 1b, 2a, 2b, 3a, 4a, 4b, 5, 6a, 7a, 8a, 8b, 9, 10, 11a, 11b, 12a, 13a, 13b, 14a, 14b, 15, 16, 17a, 19, 20, 21, 22, 23, 24, 25 **(83.8%)** |  |  | 3b, 6b, 7b, 12b, 17b, 18 **(16.2 %)** |
| Apostolidou, C. | 2015 | 1b, 2a, 2b, 3a, 4a, 4b, 5, 6a, 11b, 12a, 13a, 13b, 14a, 14b, 15, 16, 17a, 20, 21, 22, 25 **(56.8%)** |  | 1a, 8a, 8b, 9, 10, 11a, 18, 19, 23, 24 **(27%)** | 3b, 6b, 7a, 7b, 12b, 17b **(16.2%)** |
| Huguenin, G.V. | 2015 | 1b, 2a, 2b, 3a, 4a, 4b, 5, 6a, 7a, 8a, 8b, 9, 10, 11a, 11b, 12a, 12b, 13a, 13b, 14a, 14b, 15, 16, 17a, 18, 20, 21, 22, 23, 24, 25 **(83.8%)** |  | 1a, 19 **(5.4%)** | 3b, 6b, 7b, 17b **(10.8%)** |
| Rahbar, A.R. | 2015 | 1b, 2a, 2b, 3a, 4a, 4b, 5, 6a, 7a, 11b, 12a, 13a, 13b, 14a, 14b, 15, 16, 17a, 18, 21, 22, 25 **(59.5%)** |  | 1a, 8a, 8b, 9, 10, 11a, 19, 20, 23, 24 **(27 %)** | 3b, 6b, 7b, 12b, 17b **(13.5%)** |
| Dourado Grace K. Z.S. | 2015 | 1b, 2a, 2b, 3a, 4a, 4b, 5, 6a, 7a, 12a, 13a, 13b, 14a, 14b, 15, 16, 17a, 20, 21, 22, 25 **(56.8%)** |  | 1a, 8a, 8b, 9, 10, 11a, 11b, 18, 19, 23, 24 **(29.7%)** | 3b, 6b, 7b, 12b, 17b **(13.5%)** |
| Aghababaee, S.K. | 2015 | 1b, 2a, 2b, 3a, 4a, 4b, 5, 6a, 8a, 11b, 12a, 13a, 13b, 14a, 14b, 15, 16, 17a, 19, 20, 21, 22, 23, 25 **(64.9%)** | 11a, 24 **(5.4%)** | 1a, 8b, 9, 10, 12b, 18 **(16.2 %)** | 3b, 6b, 7a, 7b, 17b **(13,5%)** |
| Lee, Y.J. | 2016 | 1a, 1b, 2a, 2b, 3a, 4a, 4b, 5, 6a, 7a, 8a, 8b, 9, 10,11a, 11b, 12a, 13a, 13b, 14a, 14b, 15, 16, 17a, 19, 20, 21, 22, 23, 24, 25 **(83.8%)** |  |  | 3b, 6b, 7b, 12b, 17b, 18 **(16.2%)** |
| Chiu, H.F. | 2017 | 1b, 2a, 2b, 3a, 4a, 4b, 5, 6a, 8a, 10, 11a, 11b, 12a, 13a, 13b, 14a, 14b, 16, 17a, 20, 21, 22, 25 **(62.2%)** |  | 1a, 8b, 9, 15, 18, 19, 23, 24 **(21.6%)** | 3b, 6b, 7a, 7b, 12b, 17b **(16.2%)** |
| Martínez-López, S. | 2019 | 1a, 1b, 2a, 2b, 3a, 4a, 4b, 5, 6a, 7a, 11a, 11b, 12a, 12b, 13a, 14a, 14b, 15, 17a, 20, 21, 22, 25 **(62.2%)** |  | 8a, 8b, 9, 10, 13b, 18, 19, 23, 24 **(24.3%)** | 3b, 6b, 7b, 16, 17b **(13.5%)** |

Quality of reporting we used the CONSORT statement ^18^.

The studies were ordering by the year of publication.
